# Use of electronic health records to examine margin-to-reflex distance in patients without eyelid conditions affecting position

**DOI:** 10.1007/s10792-026-04124-5

**Published:** 2026-06-09

**Authors:** Jason W. Zhou, Fritz Gerald P. Kalaw, Bharanidharan R. Saseendrakumar, Sally L. Baxter, Catherine Y. Liu, Bobby S. Korn, Don O. Kikkawa

**Affiliations:** 1 UC San Diego Department of Ophthalmology, Department of Ophthalmic Plastic & Reconstructive Surgery, Viterbi Family Department of Ophthalmology and Shiley Eye Institute, University of California San Diego, 9415 Campus Point Drive, La Jolla, CA 92037 USA; 2 Division of Ophthalmology Informatics and Data Science, Viterbi Family Department of Ophthalmology and Shiley Eye Institute, University of California San Diego, La Jolla, CA 92037 USA; 3 Division of Biomedical Informatics, Department of Medicine, University of California San Diego, La Jolla, CA 92037 USA; 4 Department of Surgery, Division of Plastic Surgery, Department of Medicine, University of California San Diego, La Jolla, CA 92037 USA; 5 University of Maryland School of Medicine, Baltimore, MD 21201 USA

**Keywords:** Margin-to-reflex distance, Ptosis, Electronic health record, Reference eyelid measurements, Oculoplastic surgery

## Abstract

**Purpose:**

To characterize reference margin-to-reflex distance 1 (MRD1) measurements across different demographic factors, age, sex, race, and ethnicity, using a large electronic health record-derived (EHR) dataset.

**Methods:**

This was a single-institution observational, cross-sectional study of adult patients seen in an academic oculoplastics division from October 2018 to June 2023. MRD1 values were extracted from structured data fields and free-text progress notes. Patients with diagnoses known to affect MRD1 (e.g. dermatochalasis, ptosis) were excluded from the study. Univariate analysis was conducted using one-way ANOVA and post hoc testing, while multivariable analysis testing was done through a linear mixed-effects regression model.

**Results:**

A total of 6231 patients and 11,932 reference MRD1 values were analyzed. MRD1 decreased by approximately 0.2 mm (mm) per decade of life (*p* < 0.001). Female sex was associated with higher MRD1 measurements (3.38 vs 3.003, *p* < 0.001), while Asian race was associated with lower MRD1 values (2.99 vs 3.32, *p* < 0.001).

**Conclusions:**

MRD1 values vary significantly by age, sex, and race. These findings provide reference values for MRD1 within a clinical oculoplastics population accounting for demographic factors.

**Supplementary Information:**

The online version contains supplementary material available at 10.1007/s10792-026-04124-5.

## Introduction

Margin-to-reflex distances (MRDs) are measurements commonly used to evaluate eyelid position. Margin-to-reflex distance 1 (MRD1) is the distance in millimeters (mm) from the corneal light reflex to the upper eyelid margin with the patient in primary gaze [1]. Previously studies suggest that the range for normal MRD1 measurements is between + 2.5 mm and + 5.0 mm, with the upper eyelid being positioned 1–2 mm inferior to the superior limbus [1–3].

Blepharoptosis is a relatively common condition where the upper eyelid height lowers, leading to functional vision and aesthetic concerns. It is associated with a reduced MRD1 measurement. Involutional ptosis is associated with aging and diabetes [4, 5]. While the majority of studies examine MRD1 in the setting of ptosis, normative data describing measurements among those without eyelid abnormalities are relatively lacking, with only a handful of published studies reporting on this. One study with 380 participants, found that MRD1 decreased with increasing age, and that variations were seen in different ethnicities and sexes [6]. Another study in 112 patients, found that variance in normative MRD exists among ethnic groups, with White patients having greater MRD than Asians [7]. Lastly, a study of 68 patients found that Latinos and Asians have similar MRD1s, but females had higher MRD values compared to males [7]. Prior studies have been limited by smaller sample sizes, which hinder ability to robustly evaluate cross-demographic differences due to lack of statistical power.

Our study aims to characterize reference MRD1 measurements among patients without eyelid conditions known to affect eyelid position across a larger cohort. We leveraged electronic health record (EHR) data to substantially increase our sample size; thereby characterizing MRD1 variations by biological age, sex, race, and ethnicity. Establishing clinically derived reference ranges is critical in providing a standard against which to diagnose common eyelid disorders, guide surgical planning, evaluate postoperative outcomes, and understand population-level racial, ethnic, and age-related variations to help individualize care, as well as to inform future research investigating periorbital anatomy and disease.

## Methods

### Study design

This observational, cross-sectional retrospective study was conducted at a single academic institution. Chart review was performed to assess the average margin-to-reflex distance across different demographic variables: age, sex, ethnicity, and race. The data was collected from EHR (Epic Systems, Verona, WI, USA) at the University of California San Diego (UCSD) Viterbi Family Department of Ophthalmology and Shiley Eye Institute from the beginning of Epic introduction in the department, October 2018, to June 2023. The study was approved by the UCSD Institutional Review Board (IRB) (Protocol number: 807913) as an exempt protocol, and it adhered to the principles of the Declaration of Helsinki. The study was conducted in compliance with the Health Insurance Portability and Accountability Act (HIPAA).

### Study population and data collection

The inclusion criteria included all adult patients (ages 18 years and older) who were seen by UCSD Division of Ophthalmic Plastic and Reconstructive Surgery physicians during the study period, resulting in a total of 26,134 patients. The discrete variables of age, gender assigned at birth, self-reported race and ethnicity as defined by the U.S. Census Bureau [8], and diagnoses were extracted from the EHR using structured query language (SQL) from the institution’s Epic Clarity database. This data was de-identified by removing all identifiers and by calculating all necessary dates with respect to January 1st of the patients’ birth year (i.e., exact visit dates were obscured to prevent re-identification). MRD measurements for each eye were derived from structured eye exam elements in the EHR or by data extraction from free-text in the providers’ progress notes.

Patients with diagnoses of eyelid pathology known to affect MRD1 (e.g., ptosis, dermatochalasis) were excluded. A range of 2.5–5 mm was set for MRD1 as determined to be the extremes of normal by previous literature [1].

### Statistical analysis

Statistical analysis was conducted using R Studio version 4.3.3 (R Foundation for Statistical Computing, Vienna, Austria). To extract MRD1 values from free-text progress notes, regular expression pattern matching was used to identify and isolate numeric measurements recorded within the text. A random subset of 150 MRD1 snippets from free-text progress notes was manually reviewed to assess accuracy, defined as agreement in numeric value and laterality. Continuous variables such as MRD and age were summarized using means and standard deviations while categorical variables were summarized with frequencies and percentages. Univariate analyses of variations (ANOVA) in MRD1 measurements were performed with two-tailed Student’s t-tests and one-way ANOVAs, followed by Tukey’s Honestly Significant Difference test for post hoc comparisons, primarily for descriptive purposes. Assumptions underlying parametric hypothesis testing were verified before conducting the t-tests. For univariate analysis, MRD1 values were averaged across both eyes and all available measurements to generate a single representative value per patient. For multivariate analysis, linear mixed-effects models were used to account for repeated measurements across visits and inclusion of both eyes per patient. Statistical significance was defined as *p* < 0.05.

## Results

### Study cohort characteristics

In total, 6231 patients and 16,278 MRD1 measurements met eligibility criteria. Structured exam fields comprised the source of 4358 MRD1 measurements, and 11,920 were from free-text progress notes, with 4346 overlapping measurements between sources. After removal of duplicate overlapping entries, 11,932 reference MRD1s were available for inclusion and analysis. The mean (standard deviation [SD]) age was 67.2 (13.6) years (Table 1). Overall, the cohort was predominantly female (4052/6231, 65.0%), and a majority self-identified as White (4411/6231, 70.2%) and not Hispanic or Latino (5330/6231, 85.5%). Data revealed that MRD1 appears to peak in the 28–37 age group, and then consistently falls with every subsequent decade.

**Table 1:** Demographic characteristics and average Margin Reflex Distance 1 (MRD1) in the study cohort

Characteristic	Category	N (%)	Mean (SD) MRD1
Sex	Male	2178 (35%)	3.03 (1.38)
	Female	4052 (65%)	3.38 (1.28)
Age group	18–27	103 (1.7%)	3.72 (1.25)
	28–37	240 (3.9%)	4.08 (0.97)
	38–47	414 (6.7%)	3.85 (1.13)
	48–57	879 (14.2%)	3.54 (1.39)
	58–67	1565 (25.2%)	3.34 (1.33)
	68–77	1696 (27.4%)	3.2 (1.27)
	78–87	1004 (16.2%)	3.03 (1.33)
	88–97	285 (4.6%)	2.94 (1.33)
	98 +	15 (0.2%)	1.44 (1.46)
Race	White	4411 (70.8%)	3.32 (1.28)
	Black or African American	123 (2.0%)	2.75 (1.4)
	Asian	633 (10.2%)	2.99 (1.45)
	Other Race or Mixed Race	788 (12.6%)	3.27 (1.47)
	American Indian or Alaska Native	21 (0.3%)	3.29 (0.81)
	Native Hawaiian or Other Pacific Islander	20 (0.3%)	3.43 (1.33)
	Unknown	235 (3.8%)	3.77 (1.22)
Ethnicity	Not Hispanic or Latino	5330 (85.5%)	3.26 (1.32)
	Hispanic or Latino	656 (10.5%)	3.32 (1.31)
	Unknown	245 (3.9%)	3.65 (1.29)

*SD* Standard deviation

### Free-text extraction validation

In a validation subset of 150 randomly selected free-text-derived MRD1 snippets, exact agreement between extracted values and manually reviewed values was observed in 113/150 (75.3%) of cases. After application of analytic inclusion criteria, only 2/150 (1.3%) discrepant cases were retained in the final reported dataset, both of which were truncated numeric values.

### Univariate analysis results

The results of univariate ANOVA of MRD1 by sex, age, race, and ethnicity are shown in Table S1. MRD values were averaged per patient across both eyes and all available visits. Sex, race, and age group were significantly associated with changes in MRD1, while ethnicity did not show statistical significance. Post-hoc testing revealed that on average, women had higher mean MRD1 than men (3.38 vs 3.04, *p* < 0.001) and Asians had lower mean MRD1 than Whites (2.99 vs 3.32, *p* = 0.001), other or mixed race (2.99 vs 3.27, *p* = 0.028), and unknown racial subgroups (2.99 vs 3.77, *p* = 0.003).

### Multivariable analysis results

To further assess the relationship between MRD1 and demographic factors, a multivariable linear regression model was performed (Table S2).

Age at exam was a significant predictor of MRD1, and MRD1 decreases by 0.019 mm for each additional year of age, after controlling for confounders. Females and males also exhibit different upper eyelid positions, with females having an average of 0.23 mm larger than males, even after controlling for other demographic variables. And finally, consistent with the univariate analysis, Asian race remained significantly associated with lower MRD1 values (0.35 mm less) compared to White race. Neither the other racial subgroups nor ethnicity was a significant predictor of MRD1.

## Discussion

This retrospective study investigated MRD1 across key demographic factors – age, sex, race, ethnicity, within a single institution cohort. We sought to characterize MRD1 trends across different population subgroups, which is crucial for diagnosing eyelid disorders, guiding clinical decision-making, and personalizing oculoplastic care. This study found the following significant findings: 1) MRD1 decreases by 0.02 mm per year of life, 2) female sex has greater MRD1 than male sex, and 3) increased age and Asian ethnicity are predictors of MRD1 even after adjusting for confounders. In this context, these findings should be interpreted as reference measurements derived from an oculoplastics clinic population rather than population-level normative values.

The study cohort was weighted toward middle-aged and older adults and was predominantly White, reflecting the patient population typically seen in an academic oculoplastics practice. Accordingly, these demographic characteristics likely influenced the overall distribution of MRD1 values, as both increasing age and White race were associated with higher MRD1 measurements in our cohort. Overall, the average MRD1 across the entire cohort was 3.28 ± 1.32, which aligns with previous smaller prospective studies which demonstrated an average of 3.5 ± 0.8 [6].

As expected, our results showed a clear statistically significant trend between age and MRD1, with eyelid height decreasing as age increased. This is consistent with earlier research which also reported the same association using two-decade age bins with smaller sample sizes ranging from 28—163 participants [6]. In contrast, our study analyzed over 16,000 continuous age measurements with greater statistical power. Aging changes in the levator palpebrae superioris likely affect function, as well as decreases in skin elasticity and thinning of the epidermis, leading to skin laxity [9, 10]. In the present study, MRD1 decreased by 0.2 mm per decade, reinforcing the importance of age-adjusted reference values when evaluating eyelid position.

In addition to age-related effects, sex-based differences were also observed, with higher MRD1 measurements in female patients. The higher MRD1 observed in female patients may reflect differences in brow position, frontalis muscle recruitment, and periorbital soft tissue characteristics, although the underlying anatomical basis remains incompletely understood [11, 12].

Previous research has indicated racial differences in MRD1 with White patients having higher MRD1 values compared to Asians from a prospective cohort of 112 patients [13]. The present study confirms similar findings using a much larger sample size [7], with Asians having an average MRD1 of 2.99 mm, a statistically significant difference compared to Whites (3.32 mm), and the other/mixed group (3.27 mm). These differences persisted in our multivariable model even after adjusting for confounders. All ethnicities, except American Indians/Alaskan Natives, had smaller upper eyelid measurements compared to Whites, but did not reach statistical significance. Some racial subgroups (e.g., American Indian/Alaska Native and Native Hawaiian/Other Pacific Islander) had small sample sizes, thus findings in these groups should be interpreted with caution. While our study utilized the U.S. Census-derived categories for race and ethnicity; however, these categories remain broad and may conceal variations within subgroups, particularly among Asian populations (e.g., East Asian subgroups), which represent an important area for future study.

While MRD1 remains a commonly used clinical measurement, its accuracy and reliability warrant closer inspection. Rootman found that clinical MRD1 measurements taken with a ruler and digital measurements were no different from one another. The same held true for ruler versus slit lamp measurements [14]. However, both clinical and photographic measurements remain susceptible to variability from frontalis muscle recruitment. Patients may inadvertently elevate their eyebrows during examination or photography, secondarily increasing the apparent MRD1. While manual brow stabilization can mitigate this during clinical exam, this factor is more difficult to control in photographic or automated systems and may introduce additional measurement variability. When using the Volk device (Volk Optical, Inc, Mentor, OH, USA), a modified smartphone that measures MRD1 automatically, it was found to be accurate in measuring control patients, but overestimated MRD in patients with ptosis [15]. Despite this, no studies to date have examined the reliability of these measurements between different providers. In the present study, formal assessment of inter-rater reliability was not feasible due to the retrospective design, as measurements were obtained during routine clinical care without standardized repeated assessments by multiple observers. However, all measurements were performed by fellowship-trained oculoplastic surgeons within a single institution, which may reduce variability compared to more heterogeneous settings. Prospective studies with standardized measurement protocols would be needed to formally assess inter-rater reliability, which is important given the nuanced nature of the measurement, which relies on visual estimation and manual techniques. Given these concerns, there is growing interest in using alternative such as AI-driven analysis of clinical photographs [14–16], automated photogrammetric systems, auto-refractometer [16], smartphone-based photographic applications [14] to obtain objective measurements of MRD more conveniently and reliably.

Our study has several limitations. The retrospective design and oculoplastics clinic-based population limit our ability to establish true population-level normative MRD1 values. At our institution, oculoplastic specialists are the only physicians who consistently measure MRD, therefore the majority of patients referred have an orbital or eyelid diagnosis. This introduces an inherent sampling bias, as patients presenting to an oculoplastic clinic often have conditions that may directly or indirectly influence upper eyelid position. Although patients with conditions known to affect eyelid position (e.g., ptosis, dermatochalasis) were excluded, more subtle effects from underlying oculoplastic or orbital pathology cannot be excluded. These factors may systematically influence MRD1 measurements and limit generalizability to patients seen in routine comprehensive ophthalmology or primary care settings. A prospective study evaluating MRD1 in patients without oculofacial pathology would be ideal to establish true normative values. Additionally, there was a difference noted in recorded MRD1 measurements based on data entry method. Measurements from the structured Epic module (Kaleidoscope) tended to be higher than those text entries in progress notes, with a mean MRD1 of 3.62 compared with 3.15. One contributor to this difference is that the entry of discrete values into the ophthalmology exam interface requires rounding to the nearest 0.5 mm. Although measurements of less than 0.5 mm may not be clinically significant or even distinguishable, it may have reduced the precision of recorded measurements. This discrepancy may reflect natural variations in clinical practice, but ultimately, standardization of data entry methods across EHRs may be necessary to ensure accurate and precise measurements. Next, reliance on progress note free-text-extracted MRD1 measurements introduces potential for analysis and documentation-related variability, including non-standard formatting and incomplete or truncated numeric entries, as well as copy-forward errors. Additionally, averaging MRD1 measurements across visits and both eyes for univariate analyses may reduce within-patient variability; however, mixed-effects modeling in multivariable analyses accounted for repeated measurements and mitigated this limitation. Finally, while our sample size was the largest reported in the literature, the data was drawn from a single institution, possibly limiting the generalizability of these findings to other populations.

In summary, this is the first study to utilize EHRs in evaluating reference MRD1 values across broad demographic factors, using the largest clinical cohort reported to date. Future research should focus on leveraging larger, multi-center databases with more granular demographic data to expand the cohort and ensure that the reported reference MRD1 values, as well as other clinically relevant periorbital measurements, can be generalizable across diverse populations, both demographically and geographically.

## Supplementary Information

Below is the link to the electronic supplementary material.

Supplementary file1 (DOCX 14 kb)

## Data Availability

Our data was generated from Epic EHR, and was subsequently de-identified and removed of any PHI. Data can be made available at reasonable request to the corresponding author.

## Declarations

### Meeting presentation

Association for Research in Vision and Ophthalmology Annual Meeting 2025, Association for Academic Minority Physicians Annual Meeting 2023.
