# Supplementary material for: Use of electronic health records to examine margin-to-reflex distance in patients without eyelid conditions affecting position
Source: Int Ophthalmol. 2026 Jun 9;46(1):258. doi: 10.1007/s10792-026-04124-5 (PMC13249627; doi:10.1007/s10792-026-04124-5)
Supplement: Supplementary file 1 — Supplementary file1 (DOCX 14 kb) [file 10792_2026_4124_MOESM1_ESM.docx]

Table S1. Analysis of Variance (ANOVA) for Margin Reflex Distance 1 (MRD1) across demographic variables.

Supplemental Table S1 will be online-only.

|  | Df | Sum Sq | Mean Sq | F value | P-value | Post-hoc testing |
| --- | --- | --- | --- | --- | --- | --- |
| Sex | 1 | 34.50 | 34.45 | 28.905 | < 0.001 | women > men (p < 0.001) |
| Age Group | 8 | 158.20 | 19.78 | 16.594 | < 0.001 | 28-37 > 58-67, 68-77, 78-87, 88-97, 98+ (p = 0.002), 38-47 > 58-67, 68-77, 78-87, 88-97, 98+ (p = 0.007), 48-57 > 58-67 (p = 0.014), 68-77, 78-87, 88-97, 98+ (p = 0.036), 58-67 > 78-87 (p = 0.001), otherwise p < 0.001 |
| Race | 6 | 32.90 | 5.48 | 4.601 | 0.001 | asian < white (p = 0.001), asian < other / mixed (p = 0.028), asian < unknown (p = 0.003) |
| Ethnicity | 2 | 3.40 | 1.71 | 1.437 | 0.238 |  |

Table S2. Linear Mixed-Effects Regression of Mean Margin Reflex Distance 1 (MRD1) and demographic predictors.

Supplemental Table S2 will be online-only.

|  |  | Value | 95% CI | Standard Error | t-value | p-value |
| --- | --- | --- | --- | --- | --- | --- |
| Age | Age at exam | -0.019 | (-0.022, -0.015) | 0.002 | -11.834 | 0.000 |
| Sex | Sex Female | 0.229 | (0.137, 0.321) | 0.047 | 4.891 | 0.000 |
| Race | Race American Indian or Alaska Native | 0.227 | (-0.349, 0.802) | 0.293 | 0.773 | 0.440 |
|  | Race Asian | -0.347 | (0.485, -0.209) | 0.07 | -4.937 | 0.000 |
|  | Race Black or African American | -0.164 | (-0.503, 0.136) | 0.163 | -1.126 | 0.260 |
|  | Race Native Hawaiian or Other Pacific Islander | -0.355 | (-1.135, 0.424) | 0.398 | -0.893 | 0.372 |
|  | Race Other Race or Mixed Race | -0.090 | (0.231, 0.051) | 0.072 | -1.256 | 0.265 |
|  | Race Unknown | 0.132 | (-0.100, 0.364) | 0.118 | 1.114 | 0.265 |
| Ethnicity | Ethnicity Hispanic or Latino | -0.059 | (-0.209, 0.092) | 0.077 | -0.763 | 0.445 |
|  | Ethnicity Unknown | 0.130 | (-0.103, 0.363) | 0.119 | 1.095 | 0.274 |
